# Pattern and management of sports injuries presented by Lagos state athletes at the 16^th ^National Sports Festival (KADA games 2009) in Nigeria

**DOI:** 10.1186/1758-2555-2-3

**Published:** 2010-01-22

**Authors:** Oluwatoyosi BA Owoeye

**Affiliations:** 1 Department of Physiotherapy, Lagos University Teaching Hospital, PMB 12003, Lagos, Nigeria

## Abstract

**Background:**

There is a dearth of information on the epidemiology of sports injuries in Nigeria. The study was aimed at documenting sports injuries sustained by Lagos state athletes during the 16^th ^National Sports Festival (KADA Games 2009). It was also aimed at providing information on treatments offered to injured athletes.

**Methods:**

The study was carried out at Amadu Bello Stadium Complex, sporting arena of the Murtala Square and the team Lagos mini clinic. Participants were accredited Lagos state athletes who at one point in time during the games required treatment from any of the members of the medical team. Demographic data of athletes, type of injuries, body parts injured and treatment modalities used were documented and analysed using descriptive statistics.

**Results:**

Within the period of the games, a total of 140 sports injuries were documented from 132 athletes with an approximate male to female ratio of 2:1 and age ranging from 15-38 years. Most of the injuries reported by the athletes were "minor" injuries. Muscle strain was the most common type of injury (31.4%) followed by ligament sprains (22.9%). The lower extremities were the most injured body region accounting for 50% of all injuries. Over 60% of injuries presented by the athletes were from basketball, cricket, hockey, rugby and baseball. Cryotherapy was the most frequently used treatment modality, followed by bandaging and massage with anti-inflammatory gels.

**Conclusion:**

Establishing injury prevention programmes directed at the lower extremities may help reduce the risk of injuries to the lower extremities. Since cryotherapy was the most used treatment modality, it is suggested that it should be made abundantly available to the medical team preferably in forms of portable cold sprays for easy transportation and application during the games. It is also important that physiotherapists form the core of the medical team since they are trained to apply most of these treatment modalities and they also play a major role in establishing injury prevention routines. This data provides information that will be useful to both state and federal medical teams in preparing for future games.

## Introduction

The National Sports Festival (NSF) is a biennial sporting competition for young and up-coming athletes in Nigeria. The 16^th ^NSF tagged KADA '09, was held in Kaduna, a state located in the far north of Nigeria, from the 15th to 25^th ^February, 2009. Athletes participated in sports such as football, basketball, hockey, boxing, karate, cricket, chess, handball, athletics and traditional sports among several other sports. The traditional sports are local Nigerian sports which includes "Ayo" (A seed game where one with the largest collection is declared winner), "Abula" (A ball game played across the net with a wooden baton) and "Langa" (A hopping game of standing/running with one leg, where in opponents struggle to dislodge one other). The NSF was the largest sporting event ever to take place in Kaduna, the host state.

All the 37 states in Nigeria, including the Federal Capital Territory (Abuja) participated in the event. A total of 10, 708 athletes were accredited for the event with Kaduna presenting the highest number with 711 athletes while Jigawa State had the lowest number with 69 athletes [1]. Team Lagos participated in all sports events except male/female handball and football (did not qualify at the zonal eliminations) with a total of 655 athletes. Team Delta emerged winner for the 3^rd ^consecutive time while team Lagos ended up at 7^th ^position [1,2].

There is a dearth of information on the epidemiology of sports injuries in Nigeria. At present, there is no information concerning the pattern of injuries sustained by participating athletes during the 16^th ^NSF (KADA Games '09) held at Kaduna State and information on epidemiology of injuries during NSFs in Nigeria is generally lacking. The purpose of the study was therefore to investigate the pattern of sports injuries sustained by Lagos athletes during the 16^th ^NSF and the treatment modalities used in managing such injuries; thereby providing information that will help the state and others in planning for future games in terms of strategies for injury prevention and better medical coverage.

## Methods

### Description of Sports Facilities and Participants

The events occurred at the Amadu Bello Stadium Complex and the newly constructed gigantic sporting arena at the Murtala Square. The Amadu Bello Stadium Complex was the venue for most events including the opening and closing ceremonies. Participants in this study were accredited Lagos state athletes who at one point in time during the games required treatment from any of the members of the medical team. A total of 655 athletes (395 males, 260 females) were registered by the Lagos State Sports Council for the games. Participants were grouped according to their sports. Male/female rugby {50 (25 each)} had the highest number of athletes while scrabble (10) had the least number of athletes [3]. The mean age (SD) of the athletes was 23.3 (3.9) ranging from 15-38 years.

### Description of Personnel and Medical Facilities

The medical team of the Lagos state contingents had 4 doctors, 5 physiotherapists and 4 nurses. Injured athletes were treated either at game venues (on-site) or at the Team Lagos mini clinic located at the athletes' hostel (off-site). Due to low staff strength, members of the team were assigned to the following sports for on-site treatment: basketball, baseball, rugby, taekwondo, cricket, hockey, wrestling, boxing, traditional sports, judo and karate (most of which are contact sports). Each member of the medical team was assigned a sport. However, members were not restricted to their primary sports of duty; other sports were also covered as chance permitted. The head of the medical team (a sports physician) acted as the director and supervisor while a nurse matron was in charge of the Team Lagos mini-clinic located at the athletes' hostel. All members of the medical team reported at the mini-clinic in the evenings to meet and attend to injured athletes with complaints.

Members of the medical team had structured log books for assessment and documentation of treatments given to athletes both on-site and off-site. Data recorded included: demographics of athletes, type of injuries, body parts injured and treatment modalities used. For the purpose of this study, an "injury" was defined as any physical complaint sustained by an athlete newly incurred during the games and needing the attention of any member of the medical team. Injuries documented were evaluated and classified as either: (1) "minor" (injury in which an athlete was able to return to his/her game immediately after an on-site treatment or able to participate in the next game after an off-site treatment); (2) "moderate" (injury in which an athlete was unable to return to his/her game immediately after an on-site treatment or unable to participate in the next game after an off-site treatment); (3) "major" (a potentially life-threatening injury demanding immediate referral to a tertiary institution) (Table 1). Assessment and treatment for non-sport related complaints such as fever, cough, stomach ache etc from athletes and Team Lagos officials were recorded in separate log books. Most of such cases were attended to by the medical team director and nurse matron at the mini clinic.

**Table 1:** Classification of Injuries

Classification	Description	Types of Injuries
Minor	Injury in which an athlete was able to return to his/her game immediately after an on-site treatment or able to participate in the next game after an off-site treatment	Lacerations, bruises, contusions, muscle cramps/spasm, mild sprains and strains.

Moderate	Injury in which an athlete was unable to return to his/her game immediately after an on-site treatment or unable to participate in the next game(s) after an off-site treatment	Lacerations involving suturing, joint dislocations, moderate sprains/strains, small bone fractures.

Major	A potentially life-threatening injury that demanded immediate referral to a tertiary institution	Head injuries, multiple fractures of long bones, spinal cord injuries.

Injury severity and operational definitions for the study

### Data Management and Statistical Analysis

Log books were returned by all the members of the medical team and all data were compiled. Athletes' exposures were based on the number of participants officially registered by Lagos State for each sport. Injury risk (per sport and gender) was calculated as total number of injuries divided by total number of players for each sport and gender. The data were analysed using descriptive statistics of frequency, percentage and bar charts. The analysis was performed in SPSS, version 15 for Windows (Lead Technologies Inc SPSS Inc, Chicago, Illinois, USA).

## Results

From a cohort of 655 athletes (395 males and 260 females), a total of 132 athletes were attended to within the period of the games (15th to 25^th ^February, 2009) and 140 sports injuries were documented by members of the medical team. 92(65.7%) of these injuries were presented by male athletes while 48(34.3%) were presented by female athletes; giving an approximate ratio of 2:1 and an injury risk of 0.23 and 0.19 in male and female athletes respectively.

### Injuries

**Figure 1:**
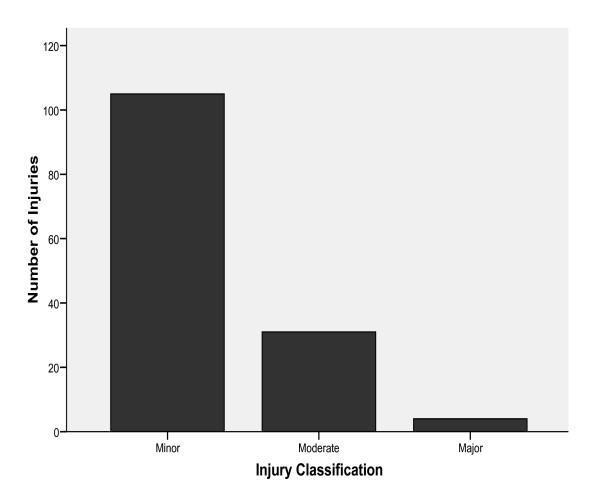
**Severity of Injuries**.

105 (75%) of the injuries reported by the athletes were classified as "minor" in severity while 31 (22.1%) and 4 (2.9%) were "moderate" and "major" respectively (Figure 1). Muscle strain was the most common type of injury (n = 44, 31.4%), followed by ligament sprain (n = 32, 22.9%) and bruises (n = 16, 11.4%) (Table 2).

**Table 2:** Types of Sports Injuries

Injury	n	%
Ligament Sprain	32	22.9
Muscle Strain	44	31.4
Muscle Spasm/Cramp	7	5.0
Laceration	14	10.0
Bruises	16	11.4
Contusion	5	3.6
Back Pathology/Pain	9	6.4
Overuse Injury	7	5.0
Dislocation	4	2.9
Fracture	2	1.4

**Total**	**140**	**100**

The ankle/foot was recorded as the most injured part of the body with 27(19.3%) presentations; followed by head/neck (n = 22, 15.7%). The elbow/forearm and groin presented the least number of injuries (Table 3).

**Table 3:** Body Parts Injured

Body Part Injured	n	%
Head/Neck	22	15.7
Shoulder/Arm	16	11.4
Elbow/Forearm	5	3.6
Wrist/Hand	18	12.9
Back	9	6.4
Grion	5	3.6
Thigh	10	7.1
Knee	19	13.6
Leg	9	6.4
Ankle/Foot	27	19.3

**Total**	**140**	**100**

Basketball (n = 22, 15.7%) presented the highest number of injuries and the highest injury risk (0.92) followed closely by Cricket (n = 19, 13.6%) with an injury risk of 0.86. Athletics, volleyball, kickboxing and kung-fu presented the least number of injuries (n = 2 in each case) (Table 4).

**Table 4:** Distribution and Risk of Injuries by Sports

Sport	n	%	Injury Risk
Basketball	22	15.7	0.92
Baseball	15	10.7	0.47
Cricket	19	13.6	0.86
Rugby	17	12.1	0.34
Hockey	18	12.9	0.50
Athletics	2	1.4	0.13
Wrestling	10	7.1	0.50
Volleyball	2	1.4	0.17
Taekwondo	9	6.4	0.41
Boxing	6	4.3	0.30
Gymnastics	3	2.1	0.25
Tradtional Sports	5	3.6	0.15
Karate	3	2.1	0.20
Kickboxing	2	1.4	0.09
Judo	5	3.6	0.28
Kung-fu	2	1.4	0.09

### Treatment

**Figure 2:**
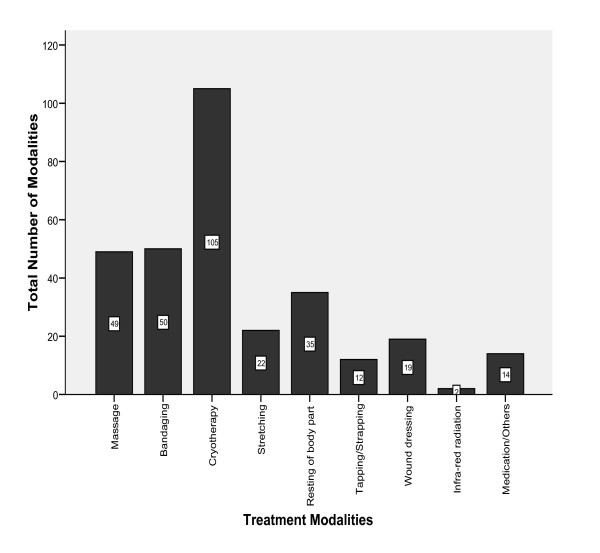
**Frequency of Usage of Treatment Modalities**.

A total of 308 separate treatments applications were rendered. Cryotherapy (n = 105) was the most frequently used treatment modality, followed by bandaging (n = 50) and massage (n = 49). Infra-red radiation therapy was recorded to have the least usage (n = 2) (Figure 2).

## Discussion

To the best of my knowledge, this study is the first to describe sports injuries sustained by athletes during a NSF in Nigeria. Even though the study presents sports injuries reported by athletes from Lagos states only, the information provided reveals the type of sports injuries to expect from a sample of athletes participating in the NSF. Furthermore, studies on the types of treatment modalities administered on Nigerian athletes during major sporting events such as the NSF were not found while searching literatures for the present study. Such data are necessary for adequate planning of medical coverage by the state and federal governments.

Similar to the profile of injuries in other studies [4-6], most of the injuries sustained during the 16^th ^NSF were minor in severity. The gender distribution (male/female) of sports injuries was found to be in the ratio 2:1 and incidence of injuries was higher in male athletes. This may be because Team Lagos had more participating male athletes than females; exposing them to a higher risk of injuries. This finding agrees with the common trend in literature that more males participate in sports than females [4-10]. Although, participation of female athletes is on the rise globally, male athletes are still much more involved in sports than females.

As in other studies [5-10], muscle strains and ligament sprains were documented as the most prevalent injuries. Although a considerably high frequency of injuries was recorded for the head/neck and the upper limb (15.7% and 27.9% respectively) the lower limb (50%) was the most injured body part. It has been generally documented that the lower extremities pose the highest number of sports injuries in sports medicine clinics [7,8] and during sports competitions [4-6,10]. The reason for a high prevalence of head/neck and upper limb injuries during the 16^th ^NSF may be because most sports involving heavy use of the upper limbs (such as basketball, cricket, baseball) and hitting of the head (boxing, taekwondo, karate) were adequately covered by members of the medical team. In this study, muscle strains were documented to be more prevalent in the neck, shoulder and arm while ligament sprains were recorded to be more prevalent in the lower limbs, especially the knee and ankle joints.

A prevalence of over 60% of injuries in basketball, baseball, cricket, rugby and hockey contradicts some other studies where athletics was reported to have the highest number of injuries [4,6,7]. It is interesting to know that athletics with a company of a few other sports had the lowest injury risk during the games. This may be because athletics and some other sports were not assigned medical personnel due to low staff strength. It is therefore possible that a few minor on-site injuries sustained by athletes in these sports might have been missed. It is also interesting to know that rugby, despite having the highest number of athletes had the lowest injury risk among the team sports. Reason for this may be because Team Lagos lost out at the preliminary stages of both male and female rugby.

The 308 applications of treatment modalities on injuries sustained within this time period of the games may not be a true reflection of the total number of applications; sometimes treatments rendered might not have been recorded as indicated by Thompson and Ratecliffe [6]. Cryotherapy, bandaging and massage were the most frequently used treatment modalities during the games. This is in accordance with a study by Jelsma et al [10]. However, therapeutic ultrasound, transcutenous electrical nerve stimulator (TENS), exercise therapy and massage were documented as more frequently used treatment modalities over cryotherapy in other studies [4,9]. There may not be any bases for comparison in this case; these studies focused on injuries treated off-site at a polyclinic and the physiotherapy department of a sports medicine centre where several other sophisticated modalities were available. The present study investigated treatment interventions both on-site and off-site and most of the injuries were "minor" acute injuries that required cryotherapy as first aid on-site intervention. However, availability of a therapeutic ultrasound machine and some other physiotherapy modalities such as TENS and tapping materials would have helped in better off-site management of some "minor" injuries sustained by key athletes who were indispensable players in some team sports.

A few treatments in the category of "others" were administered mostly on "moderate" injuries and these included prescriptions of pain medications/injections and suturing of deep lacerations.

## Conclusion

Most of the the injuries sustained were "minor" muscle strains and ligament sprains. Incidence of sports injuries was highest in basketball and majority of injuries were to the lower extremities. Establishing injury prevention programmes directed at the lower extremities may help reduce the risk of injuries to the lower extremities.

Since most of the treatments rendered to athletes were cryotherapy, bandaging and application of anti-inflammatory gels through massage, it is suggested these modalities be made abundantly available to the medical team for subsequent games. Provision of cryotherapy gadgets in forms of portable cold sprays should be a preference for easy transportation and application. It is also important that physiotherapists form the core of the medical team since they are trained to apply most of these treatment modalities and they also play a major role in establishing injury prevention routines. This study provides information that will be useful to both state and federal medical teams in preparing for future games.

## Competing interests

The author declares that he has no competing interests.
